# High burden of breast cancer in Belgium: recent trends in incidence (1999-2006) and historical trends in mortality (1954-2006)

**DOI:** 10.1186/0778-7367-69-2

**Published:** 2011-10-24

**Authors:** Françoise Renard, Liesbet Van Eycken, Marc Arbyn

**Affiliations:** 1 Belgian Cancer Registry, Rue Royale 215, B-1210 Brussels, Belgium; 2 Unit of Cancer Epidemiology, Scientific Institute of Public Health, Rue Juliette Wytsmanstraat 14, B-1050 Brussels, Belgium

## Abstract

**Introduction:**

In Belgium, breast cancer mortality has been monitored since 1954, whereas cancer incidence data have only been made available for a few years. In this article we update historical trends of breast cancer mortality and describe the recent breast cancer incidence.

**Methods:**

Incidence data were extracted from the Belgium Cancer Registry from 2004 to 2006 for the Walloon and Brussels Regions and Belgium, and from 1999 to 2006 for the Flemish Region. The Directorate-general Statistics and Economic information provided the mortality data for the years 1954-1999 and 2004. The regional authorities of the Flemish and Brussels Regions provided the mortality data for the years 2000-2003 and 2005-2006.

**Results:**

In 2004, the World age-standardised breast cancer incidence for the whole of Belgium was 110 per 100, 000 person-years for all ages; and 172, 390 and 345 per 100, 000 person-years for the 35-49, 50-69, and 70+ age groups, respectively. The incidence rate was slightly higher in each age group in the Brussels Region. In Flanders, where the incidence could be observed during a longer period, an increase was observed until 2003 in the 50-69 age group, followed by a decrease. To the contrary, in the oldest age group, incidence continued to rise over the whole period, whereas no change in incidence was observed between 1999 and 2006 in the 35-49 age group.

Mortality increased until the late 1980s and afterwards decreased in all regions and in age groups younger than 70. In women of 70 years and older, the decline began later.

**Conclusions:**

The burden of breast cancer in Belgium is very high. In 2004, Belgium ranked first for the age-standardised incidence rate in Europe for all ages combined and in the 35-49 and 50-69 age groups. The impact of the known risk factors and of mammographic screening should be further studied. The mortality rate in Belgium ranked lower than incidence, suggesting favourable survival. Plausible explanations for the discrepancy between incidence and mortality are discussed.

## Introduction

Breast cancer incidence has shown a generalised increase over the past century, with higher levels in industrialised compared to non-industrialised countries [1], mainly resulting from

major lifestyle changes favouring breast cancer, such as lower parity, postponed childbearing, reduced breast feeding, sedentariness, and obesity [2,3]. Since the last decades of the 20^th ^century, the use of hormonal replacement therapy (HRT) by women entering menopause contributed to the increase of the incidence [4,5]. At the same time, the introduction of mammographic screening in women of about the same age has led to an apparent increase in incidence because of earlier detection and some extent of overdiagnosis.

Before 2004, the International Agency for Research on Cancer (IARC) estimates of breast cancer incidence in Belgium [6] were based on incidence in neighbouring countries adjusted for mortality in Belgium. These estimates were very high, but no one knew whether this statistical modelling reflected the reality. After an initial period where cancer registration was only done in the Flemish Region [7], the Belgian Cancer Registry has been recently set up, producing real data [8], which fills a considerable gap in Europe. Recent comparisons between countries made by the IARC with the true Belgian data for 2005 indicate that Belgium belongs to the countries with the highest incidence of breast cancer in the world [9].

The age-adjusted mortality rate is a key indicator to monitor the progress in breast cancer control [10,11]. In contrast with the steady rise of incidence, the increase in breast cancer mortality in most industrial countries was interrupted in the late 1980s and was followed by a flattening or even a decrease [1,12,13]. In this paper, we update previous analyses on breast cancer mortality trends [14] and compare rates between the Flemish, Walloon and Brussels Regions for the period of which regional data are available.

Belgian incidence and mortality rates by age groups are subsequently compared with those of other European countries.

## Materials and methods

### Data sources

Belgium is a federal state divided into three regions (the Flemish, Walloon and Brussels Regions), with a complex distribution of healthcare organisation and data collection responsibilities. This results in asymmetric data availability [15].

#### Incidence

A national Cancer Registry has been recently set up. Methods of data collection have been described elsewhere [16,17]. For this study we extracted breast cancer incidence data from the Belgian Cancer Registry by year, region and five-year age groups. Data are available from 1999 to 2006 for the Flemish Region and from 2004 to 2006 for the Brussels and Walloon Regions, and thus for the whole country. Tumour pathological size (pT) according to TNM Classifications 5 and 6 were also extracted [18,19], with a proportional reallocation of the pTx. The incidence data in the Flemish Region in 1999-2002 were published by the IARC in "Cancer Incidence in five continents" [20], which attests for a good level of quality. The data for the whole country should be published in the next edition.

#### Mortality

The Directorate-general Statistics and Economic information (DGSEI), formerly known as the National Institute of Statistics (NIS-INS) [21] provided breast cancer mortality data by calendar year and five-year age group for the years 1954 until 1999 (included) and 2004 for the whole country. Regional data were available for 1969-99 and 2004. The publication of mortality data in the Walloon Region has run into delays, resulting in a gap in available data for 2000-2003, the year 2005 being in preparation at the time this report was written. The mortality data from the Flemish and Brussels Region for the years 2000-2003 and 2005-2006 were obtained directly from the respective regional authorities [22,23].

#### Population

The midyear population data (females only) by year, region, and five-year age group were computed from data provided by the DGSEI.

### Calculation and analysis

Crude rates (CR) were calculated by dividing the number of new cases in a given year by the total female population in this year and expressed per 100, 000 person-years. Age-standardised rates per 100, 000 person-years (WSR) were calculated for incidence and mortality using the world standard population [24]. Truncated standardised rates as described by Jensen [25] were calculated for three age groups, namely 35-49, 50-69 and ≥70 years of age.

The average incidence rates in the years 2004-2006 for the three regions were compared by age group and pT.

We subsequently compared the Belgian age-standardised breast cancer incidence and mortality rates of 2004, for all ages together and by age group, with those published by Héry et al. [26] in 29 other European countries in the period spanning 2000-2005 (depending on the last available year). In Hery's study, breast cancer mortality data were extracted from the WHO mortality database [27], which in July 2007 (date of last access) did not yet include the 2004 mortality rates for Belgium, and incidence data were extracted from volume VIII of "Cancer Incidence in Five Continents" [28] and the Eurocim Database [29]; which did not contain data for Belgium. Age-standardised rates (WSR) were computed in the article of Héry et al. for all ages and for the age groups 35-49, 50-69 and ≥70. We completed those rates with the Belgian rates and presented them graphically.

### Trend analyses

#### Graphical presentation of trends

We plotted the standardised incidence and mortality rates for all ages and each age group by calendar year for the whole country. At the level of the regions, mortality rates were aggregated by five-year periods in order to smooth out the variability due to the small numbers.

We also plotted the age-specific mortality rates by birth cohorts. The birth cohorts include women born in the same period so they have been exposed to common risk factors throughout their lives at the same age. This way of presenting the data can give insights into phenomena affecting specific generations. Cohorts are defined by subtracting the first year of each five-year age category from the first year of each five-year period around the death date. Since the age groups and the calendar periods both span five years, the birth cohorts are ten years wide. Successive birth cohorts overlap partly and are usually indicated by their central year [30,31].

#### Join point analysis

Temporal patterns of rates across different time periods for each age group were analysed by calculating annual percentage changes (APCs) and the 95% confidence intervals (95% CIs) for the disease rates, with log-linear Poisson models, using "join points". This method, as well as a computer software, were developed by the US National Cancer Institute [32,33], and have been used in several studies to identify temporal patterns in death rates [12,34]. We used the Join Point Regression Software, version 3.4.3 [35].

## Results

### Breast cancer incidence

The absolute numbers, the crude and age-standardised incidence rates by region and age group are presented in Table 1 for the available years. The age-standardised rates varied between 153 and 198 per 100, 000 women-year in the 35 to 49 years age group, between 322 and 437 per 100, 000 in women aged 50-69 years, and between 325 and 417 per 100, 000 in the oldest group.

**Table 1:** Breast cancer incidence by region and age group, Belgium, 1999-2006

		Flemish Region			Walloon Region			Brussels Region			Belgium		
**Year**	**Age**	**N**	**CR**	**WSR**	**N**	**CR**	**WSR**	**N**	**CR**	**WSR**	**N**	**CR**	**WSR**

1999	35-49	1014	153, 4	158, 2	*NA*	*NA*	*NA*	*NA*	*NA*	*NA*	*NA*	*NA*	*NA*
	50-69	2247	332, 0	332, 4	*NA*	*NA*	*NA*	*NA*	*NA*	*NA*	*NA*	*NA*	*NA*
	70+	1293	318, 7	325, 8	*NA*	*NA*	*NA*	*NA*	*NA*	*NA*	*NA*	*NA*	*NA*
	**all ages**	4676	155, 5	98, 4	*NA*	*NA*	*NA*	*NA*	*NA*	*NA*	*NA*	*NA*	*NA*

2000	35-49	1060	158, 5	163, 2	*NA*	*NA*	*NA*	*NA*	*NA*	*NA*	*NA*	*NA*	*NA*
	50-69	2398	352, 4	353, 2	*NA*	*NA*	*NA*	*NA*	*NA*	*NA*	*NA*	*NA*	*NA*
	70+	1341	323, 4	322, 0	*NA*	*NA*	*NA*	*NA*	*NA*	*NA*	*NA*	*NA*	*NA*
	**all ages**	4909	162, 9	102, 5	*NA*	*NA*	*NA*	*NA*	*NA*	*NA*	*NA*	*NA*	*NA*

2001	35-49	1157	171, 3	174, 9	*NA*	*NA*	*NA*	*NA*	*NA*	*NA*	*NA*	*NA*	*NA*
	50-69	2671	390, 8	388, 4	*NA*	*NA*	*NA*	*NA*	*NA*	*NA*	*NA*	*NA*	*NA*
	70+	1411	332, 2	333, 7	*NA*	*NA*	*NA*	*NA*	*NA*	*NA*	*NA*	*NA*	*NA*
	**all ages**	5341	176, 7	110, 3	*NA*	*NA*	*NA*	*NA*	*NA*	*NA*	*NA*	*NA*	*NA*

2002	35-49	1029	151, 2	153, 3	*NA*	*NA*	*NA*	*NA*	*NA*	*NA*	*NA*	*NA*	*NA*
	50-69	2751	400, 2	398, 7	*NA*	*NA*	*NA*	*NA*	*NA*	*NA*	*NA*	*NA*	*NA*
	70+	1394	320, 8	319, 6	*NA*	*NA*	*NA*	*NA*	*NA*	*NA*	*NA*	*NA*	*NA*
	**all ages**	5280	174, 1	107, 4	*NA*	*NA*	*NA*	*NA*	*NA*	*NA*	*NA*	*NA*	*NA*

2003	35-49	1146	167, 5	169, 4	*NA*	*NA*	*NA*	*NA*	*NA*	*NA*	*NA*	*NA*	*NA*
	50-69	2825	407, 5	406, 7	*NA*	*NA*	*NA*	*NA*	*NA*	*NA*	*NA*	*NA*	*NA*
	70+	1493	337, 3	336, 5	*NA*	*NA*	*NA*	*NA*	*NA*	*NA*	*NA*	*NA*	*NA*
	**all ages**	5572	183, 0	112, 6	*NA*	*NA*	*NA*	*NA*	*NA*	*NA*	*NA*	*NA*	*NA*

2004	35-49	1128	164, 6	165, 9	672	179, 0	178, 9	194	184, 0	189, 8	1994	171, 0	172, 1
	50-69	2583	370, 8	370, 8	1577	414, 6	413, 8	439	436, 6	437, 1	4599	390, 5	390, 2
	70+	1540	345, 2	342, 1	884	339, 3	342, 9	276	362, 8	376, 9	2700	344, 9	345, 0
	**all ages**	5339	175, 1	105, 9	3178	182, 6	114, 8	928	178, 6	122, 2	9445	177, 9	110, 2

2005	35-49	1178	171, 5	171, 2	647	172, 3	170, 9	202	190, 0	193, 7	2027	173, 4	173, 2
	50-69	2574	365, 1	364, 4	1434	370, 8	374, 4	365	360, 3	358, 9	4373	366, 6	366, 9
	70+	1639	360, 5	354, 1	928	353, 7	353, 8	288	382, 6	395, 1	2855	360, 4	357, 1
	**all ages**	5500	179, 5	107, 0	3061	175, 1	107, 9	870	166, 3	110, 3	9431	176, 8	107, 5

2006	35-49	1158	168, 3	167, 0	649	172, 7	171, 3	198	184, 0	189, 5	2005	171, 1	170, 4
	50-69	2570	359, 4	360, 2	1459	370, 5	375, 2	419	410, 3	408, 6	4448	367, 3	368, 8
	70+	1684	364, 6	361, 2	883	335, 8	342, 6	296	396, 7	417, 4	2863	358, 1	359, 6
	**all ages**	5511	178, 9	105, 5	3045	173, 3	107, 9	933	176, 3	119, 2	9489	176, 8	107, 4

	35-49		168, 1	168, 1		174, 7	173, 7		186, 0	191, 0		171, 8	171, 9
average rates	50-69		365, 0	365, 1		385, 0	387, 5		402, 3	401, 5		374, 7	375, 2
2004-2006	70+		356, 9	352, 5		342, 9	346, 5		380, 6	396, 2		354, 5	353, 9
	**all ages**		177, 8	106, 1		177, 0	110, 2		173, 7	117, 2		177, 2	108, 3

#### Regional differences

The age-standardised incidence rate in 2004-2006 was 10-12% higher in Brussels than in the Flemish Region for all age groups (Table 1). The rate in the Walloon region was intermediate, except for the oldest age group, where it was equal to that in the Flemish Region.

**Figure 1:**
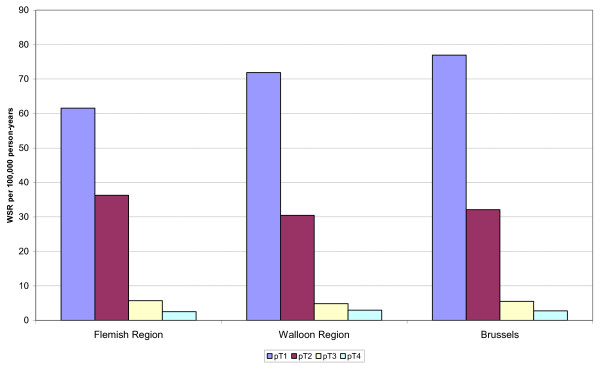
Rate of breast cancer according to the pathological T categories (pT*) by region, Belgium, 2004-2006. *The pTx (tumours of unknown size) have been proportionnaly reallocated to each pT category.

After proportional reallocation of the pTx (unknown tumour sizes), the incidence of pT1 tumours was highest in Brussels (77 per 100, 000), versus 72 per 100, 000 in Wallonia and 62 per 100, 000 in the Flemish Region (Figure 1).

Among the tumours with a known pT category, the proportion of small size tumours (pT1) was slightly higher in Brussels (62.8%) than in Wallonia (60.5%) and in Flanders (56.4%). The proportion of more advanced tumours was quite similar between the regions.

#### Time trends

**Figure 2:**
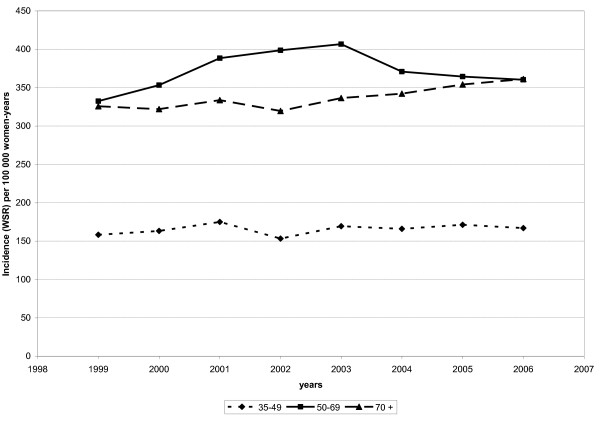
**Evolution of breast cancer incidence by age group, Flemish Region (Belgium, 1999-2006)**. Age-standardised incidence rate (WSR) per 100, 000 women-years.

Eight consecutive years of incidence data are currently available for the Flemish Region (Figure 2). While a quite stable incidence rate was observed in the premenopausal group (35-49 years), two phases were distinguished in women aged 50-69 years: a first increase from 1999-2003 (APC = 5.4%) and then a sharp decrease from 2003 to 2006 (APC = -4%). In the oldest age group, a steady increase was observed over the whole period (APC = 1.5%).

#### Comparison with twenty-four other European countries in 2000-2005

**Figure 3:**
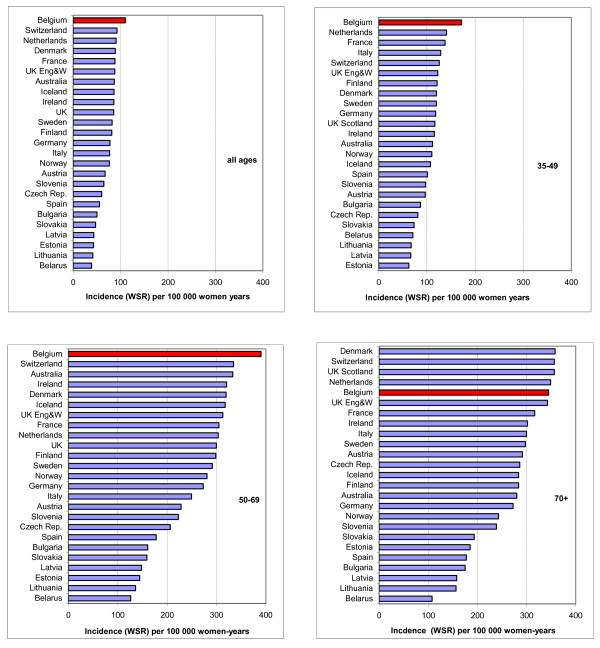
**Breast cancer incidence by age group in 2000-2005 in 25 European countries**. Age-standardised rate (WSR) for all ages (top left), 35-49 years (top right), 50-69 (bottom left) and 70 + (bottom right). Belgian rates are coloured red. Adapted from Hery *et al. *[36].

Figure 3 shows the age-standardised incidence rates (WSR) for Belgium and twenty-four other European countries sorted by decreasing incidence in 2000-2005, (depending on the last available data). Rates for the other countries were published by Héry et al [36]. The range of incidence within all the countries was 39-110 per 100, 000 women-years. Belgium ranked first for all ages together, with an incidence rate of 110 per 100, 000. This is 42% above the median rate, and 19% above the second ranking country (Switzerland). In the 35-49 year age group the rate was 172 per 100, 000; this was 54% higher than the median rate and 22% higher than the second country (The Netherlands). In the 50-69 year age group the rate was 380 per 100, 000, which was 39% higher than the median and 17% higher than the second country (Switzerland). In the age group 70+, Belgium ranked fifth with a rate of 345 per 100, 000, which was 21% above the median rate.

## Breast cancer mortality trends

### Whole of Belgium

#### All ages

**Figure 4:**
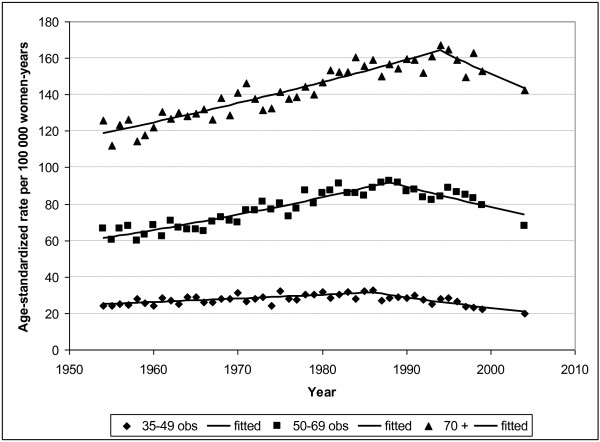
**Breast cancer mortality by age group, Belgium, 1954-2006**. Observed (points) and fitted (lines) age-standardised mortality rates (WSR) per 100, 000 women-years in the three age groups 35-49, 50-69, and ≥70 years, respectively.

Figure 4 shows the observed and fitted mortality rates in Belgium from 1954 to 1999 and for 2004. A first break point can be identified in 1986 [1980-1989] and a second one in 1996 [1993-1998]. The annual percentage changes (APC) were respectively 0.98% [0.9%-1.1%], -0.5% [-1.3%;0.2%] and -2.6% [-3.6%;-1.5%] for the periods 1954-1986, 1986-1996, and 1996-2004.

#### Changes in mortality rates by age group, Belgium

A similar trend can be observed in the three age groups, with an initial increase followed by a marked decrease in mortality rates (Table 2).

**Table 2:** Join points and annual percentage changes (APC) in mortality rates and corresponding confidence intervals (CI) in each period by age group, Belgium 1954-2004

Age group	Period	Join points [CI]	APC [CI]
All ages	1954-1986	1986 [1980-1989]	0.98 * [0.9;1.1]
	1986-1996	1996 [1993-1998]	- 0.5 [-1.3;0.2]
	1996-2004	/	- 2.6* [-3.6;-1.5]

35-49 years	1954-1986	1986 [1983 - 1991]	0.7%* [0.5-1.0]
	1986-2004	/	-2.2%* [-3.0; -1.5]

50-69 years	1954-1988	1988 [1986 - 1991]	1.2%* [1.0; 1.4]
	1988-2004	/	-1.3%* [-2.0; -0.7]

> = 70 years	1954-1994	1994 [1992-1996]	0.8%* [0.7; 0.9]
	1994-2004	/	-1.4%* [-2.2; -0.5]

*Statistically different from 0.

The breakpoint was 1986 [1983-1991] for younger women, 1988 [1986-1991] for women aged 50-69, and 1994 [1992-1996] for older women. The annual percentage of change in the second period was larger for the youngest women (NS).

### Changes in age-specific mortality rates by period and birth cohort, Belgium

**Figure 5:**
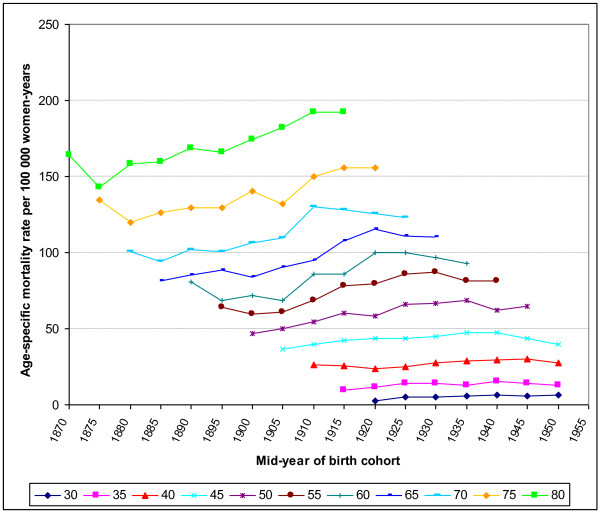
**Age-specific breast cancer mortality rates in the successive birth cohorts (1870-1950), Belgium**.

Figure 5 shows the changes in the age-specific mortality rates in the successive birth cohorts. With the exception of the oldest women, for whom the increase is quite continuous, one can see sharp increases in the age-specific mortality rates for the cohorts of women born in the first decade of the 20^th ^century (midpoints 1900 and 1905). After this continuously increasing mortality rate in successive cohorts born in the late 19^th ^and the first decades of the 20^th ^century, we discern a stabilisation or decrease that starts for older age groups in cohorts born earlier, indicating rather a period-effect situated near 1985. For instance, the change in the brown curve, which represents the women aged 55-59, occurs for the cohort born in 1930 [1925-1934], indicating something that occurred around the calendar period 1985-1989; the change in the blue curve, which represents the women aged 55-59, occurs for the cohort born in 1920 [1915-1924], indicating something occurring in the same calendar period 1985-1989.

### Mortality by region

**Figure 6:**
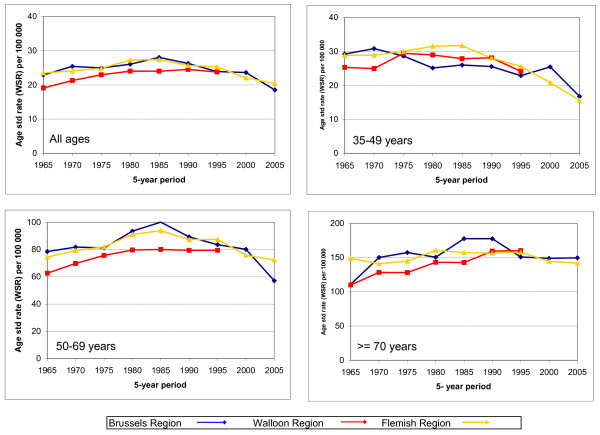
**Standardised mortality rate trends by region, for all ages (upper left), 35-49 years (upper right), 50-69 years (bottom left) and > = 70 (bottom right)**.

The age-standardised mortality rate was lower in Wallonia than in the two other regions for all ages together and in women above 50 but not in younger women (Figure 6).

The mortality rates in Brussels and Flanders decreased since the period 1985-1990, while they remained stable in Wallonia. However, it must be noted that no recent data were available for this region.

The decrease in the mortality rate in young women began earlier in Brussels than in the other regions.

### Breast cancer mortality in Belgium compared with other European countries in 2001-2004

**Figure 7:**
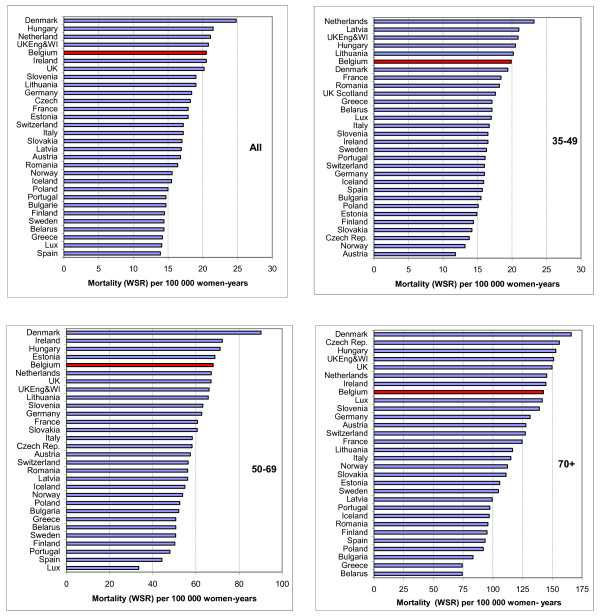
**Breast cancer mortality by age group in 2001-2004 in 30 European countries**. Age-standardised rate (WSR) for all ages (top left), 35-49 years (top right), 50-69 years (bottom left) and 70+ (bottom right). Belgian rates are coloured red. Adapted from Hery *et al. *[36].

Figure 7 shows the standardised mortality rates for thirty European countries in the years 2001-2004 (depending on the last available data).

The mortality rates in these countries range from 13.9 per 100, 000 in Spain to 24.8 per 100, 000 in Denmark. Belgium ranks fifth for all ages together, with a mortality rate of 20.5 per 100, 000. In the 35-49 age group, the Belgian rate was sixth at 19.9 per 100, 000. In the 50-69 age group the Belgian rate was fifth at 68.0 per 100.000, while in the 70+ age group the Belgian rate was eighth at 142.6 per 100, 000.

## Discussion

This study integrates all available mortality and incidence data in order to describe the current burden and the trends of breast cancer in Belgium. The incidence rate in Belgium is the highest in Europe, whereas the mortality rate ranks fifth. The mortality rate has declined in all age groups and all regions since the late 1980s, whereas the incidence seems to have continued to increase until 2003, and then stabilised and even decreased in the post-menopausal age group (50-69 years).

### Strengths and limitations of the study

In Belgium, mortality data have been available for more than fifty years. Still, the cause of death reported in mortality statistics can be of limited reliability. However, while the quality of the data on causes of death in Belgium is considered moderate by the WHO [37], the certification of deaths specifically attributed to breast cancer is considered rather reliable.

The very high rate of incidence could suggest a registration bias. Indeed, the new Cancer Registry has only recently started to register cancer cases in the Brussels and Walloon regions. During the first years of registration, it is likely that some prevalent cases are included as incident cases. Therefore, all the available pathology lab reports for the years 2004-2006 were carefully reviewed by the Cancer Registry's staff and the inclusion of prevalent or unconfirmed cases was estimated to be less than 3%. Moreover, the use of an unambiguous identifier in the registration of cancer cases (the national identification number used by the social security administration) avoids duplicate registration of the same patient. Therefore, over-registration can be considered limited.

A long-term trend analysis could only be performed for mortality, since there were no reliable incidence data before 1999 for the Flemish Region and before 2004 for the Walloon and Brussels Region.

At this stage, we limited the cohort analysis to a graphical presentation and did not perform age-cohort-period (ACP) modelling of the mortality trends. A prior ACP analysis identified a significant increasing cohort effect for post-menopausal women (≥50 years) for generations born between 1900 and 1925 (slope: 1.2%, 95% CI: 0.6-1.9%), whereas the effect was less clear in premenopausal women (slope: -1.0%; 95% CI: -1.6; -0.4%) [14]. This model should be updated with the new available mortality data.

### Current state of incidence and mortality rates

The incidence rate of breast cancer in Belgium in 2004 was the highest in Europe, for all ages together and for the 35-49 and 50-69 age groups. The excess of incidence was 42% above the median rate, and 19% above the rate of the 2^nd ^ranking country (Switzerland). However, at the same time, the mortality rate ranked fifth in Europe, being situated in the highest quartile of the European rates.

The very high incidence rate is most likely the result of several risk factors and interventions. As we explained in the introduction, decline in fertility, postponing childbearing and use of HRT are all associated with a true increase of breast cancer risk, while mammographic screening only induces an apparent rise in the incidence figures by enhancing the detection rate. Fertility indices declined continuously in the second half of the 20^th ^century in all European countries and the USA. In Belgium, the overall fertility rate fell from 2.6 children per woman in 1965 to 1.5 in 1985. From 1975 to 1995, the Belgian fertility index was quite low among EU 15 countries [38]. Childbearing has also been postponed, with the peak shifting from 24 years to 29 years between 1965 and 2000 [39]. The use of HRT is another risk factor for breast cancer. In Flanders, it was estimated to reach 20% in women of 50-69 years in 2001 [40], and could even have been higher in the other regions [41]. In The Netherlands, the rate of HRT use in menopausal women was only 13% in 1997 [42], while in France it was estimated to be 28% [43]. To our knowledge, no systematic comparison of the percentage and the type of HRT use in the European countries has been done yet, and this could be a topic for further study. The attributable fraction of the breast cancer incidence in the 50-69 years age group due to HRT use in Flanders has been put at 11% for the cancers diagnosed in 2003 [40]. The aggressiveness of HRT-induced tumours is still controversial, with old studies showing a higher proportion of localised tumours and more favourable biological features in women having had HRT [44,45], whereas a recent publication on the follow-up of the women included in the WHI study rather suggests that HRT-related tumours are more aggressive [46].

Mammographic screening produces an apparent increase in the incidence in at least two ways, namely, an advance in diagnosis (lead time bias), and the detection of slowly or non-progressive tumours, that would never have surfaced clinically (over-diagnosis) [47]. In Belgium, some opportunistic (as opposed to organised) mammographic screening began in the late 1980s, but its coverage achieved only 38% in 1999-2000. A nationwide organised screening programme was set up in 2001, while some opportunistic screening continued besides it. The overall mammographic coverage (defined as the proportion of women aged 50-69 having had a mammography over the last two years) was 59% in 2005-2006, with 28% in organised and 31% in opportunistic screening [48]. Although this coverage is not that high, it can lead to an inflated detection rate if screening sensitivity is very high.

Information on historical changes in the prevalence of other risk factors, such as obesity and sedentariness, was not available. Moreover, a large number of the etiological factors of breast cancer remain unknown.

The discrepancy between the incidence and mortality rankings suggests that some part of the excess in incidence is due to weakly aggressive tumours. This could reflect some inflation of the incidence due to the screening, since screening tends to detect some small tumours with low potential of malignancy. Since over-diagnosing small and indolent tumours can affect women's quality of life, with no impact on the mortality, there is a real need to evaluate accurately the performance indicators of both organised and opportunistic screening.

Further research should focus on estimation of the attributable fraction of all known risk factors, including a comparison between countries where information on risk factors, screening and cancer treatment is available.

The incidence rate was higher in all age groups in Brussels than in the other two regions, with a more favourable distribution of the stages in Brussels than in the Flemish Region. Indeed, while the overall coverage of screening was quite similar between the regions, the distribution of the type of screening was very different between them, with a ratio organised/opportunistic screening of 2.0, 0.2 and 0.2 respectively in the Flemish, Brussels and Walloon regions. The hypothesis of having some degree of overdiagnosis in Brussels should be examined. In any event, caution is needed in interpreting the observed differences in the stage distribution, since about 20% of the stages remain unknown.

### Trends

As in many other countries [12], we observe a strong increase in the mortality rates until the end of the 1980s, followed by a decline. The period of increase (1954-1986) definitely reflects an increase in incidence, since an increase in case-fatality rate over time seems very unlikely. The subsequent decline in mortality (after 1986) most probably corresponds to an improvement in survival rather than a decrease in incidence, as many risk factors of incidence continued to increase until the end of the century. An improvement in survival could result from several causes, such as better treatments (use of oestrogen-antagonists, better chemotherapeutic schemes, introduction of guidelines, and adherence to these guidelines), earlier diagnosis with down-staging resulting from an increased awareness of the disease and the possibilities of treatment, and mammographic screening. The decline in breast cancer mortality started before the implementation of nationwide breast cancer screening (2001) and also reached unscreened age groups; the screening probably cannot be expected to have a mortality-reducing effect before the end of the first decade of 2000, and its relative contribution to the decline in mortality observed since 1986 is likely to be low.

The decrease in mortality rates in older women was observed later, suggesting less efficient treatment schemes in this age group. This could also be explained by a delay in the mortality of women in younger age groups.

The birth-cohort analysis shows a peak in mortality for the women born between the years 1905 and 1920 suggesting a major change in the reproductive pattern at this time. This finding should be further studied.

The incidence figures could be followed for an 8-year period in the Flemish Region; a two-phase pattern was clearly observed in the 50-69-year-old, with a sharp increase until 2003 followed by a decrease. This phenomenon was interpreted as resulting from a drastic decline in HRT use [40] resulting from the publication of two large studies showing the role of those hormones in the development of breast cancer [4,5]. Similar declines in breast cancer and HRT use have been described in many other countries [43,49-52].

### Conclusions and recommendations

Both the incidence and mortality of breast cancer in Belgium are high, confirming breast cancer as a serious public health problem. The high incidence of breast cancer in Belgium results from a combination of factors, such as low fertility indices and high use of HRT, coinciding with screening effects. However, the large excess of incidence compared with the rest of Europe is not translated to mortality, where Belgium occupies the 5^th ^place in Europe. Plausible explanations for the discrepancy between incidence and mortality rankings can be effectiveness of treatment, and an inflation of the number of weakly aggressive tumours detected by screening.

It should be investigated whether differences in screening strategies could explain the regional variation in incidence.

The decreasing trend in mortality since the mid-1980s comes too early to be attributed to screening and is mostly due to improved treatment, improved access to treatment, and a better awareness of the disease.

Because they contribute to evaluating health policies, statistics on causes of death are invaluable. Filling in the gaps in the publication of Wallonia's mortality statistics is indispensable.

The high incidence of breast cancer in Belgium requires further research using analytical epidemiological methods involving individual data. This research should focus on an accurate evaluation of all the screening strategies applied (opportunistic and organised) as well as calculation of the attributable fraction of all known risk factors in Belgium and modelling of the different risk factors in Europe.

### Conflict of interest statement

The authors declare that they have no competing interests.

## Authors' contributions

FR carried out the data analysis, participated in the conception of the study and drafted the manuscript; LV coordinated the collection of incidence data, participated in the interpretation of the results and critically revised the manuscript; MA conceived the study and the design, participated in the analysis and interpretation of the results and critically reviewed the manuscript. All authors have read and approved the final manuscript.
